# Low rate of subsequent surgery and serious complications following intra-articular steroid injection for base of thumb osteoarthritis: national cohort analysis

**DOI:** 10.1093/rheumatology/keaa925

**Published:** 2021-01-07

**Authors:** Jennifer C E Lane, Richard S Craig, Jonathan L Rees, Matthew D Gardiner, Abigail V Shaw, Michelle Spiteri, Rachel Kuo, Benjamin F Dean, Jane Green, Daniel Prieto-Alhambra, Dominic Furniss

**Affiliations:** 1 Oxford NIHR Musculoskeletal Biomedical Research Unit, Nuffield Department of Orthopaedics, Rheumatology and Musculoskeletal Sciences, Nuffield Orthopaedic Centre, University of Oxford, Oxford; 2 Department of Plastic Surgery, Wexham Park Hospital, Slough; 3 Department of Hand Surgery, Nuffield Orthopaedic Centre, Windmill Road; 4 Cancer Epidemiology Unit, Nuffield Department of Population Health, University of Oxford; 5 Department of Plastic and Reconstructive Surgery, Nuffield Orthopaedic Centre, Universiy of Oxford, Oxford, UK

**Keywords:** osteoarthritis, thumb, surgical procedures, intra-articular injection, complications/adverse events

## Abstract

**Objectives:**

Intra-articular steroid injection is commonly used to treat base of thumb osteoarthritis (BTOA), despite a lack of large-scale data on safety and effectiveness. We estimate the incidence of serious complications and further procedures following BTOA injection, including the risk of post-operative serious surgical site infection for subsequent operative intervention.

**Methods:**

Hospital Episode Statistics data linked to mortality records from 1 April 1998 to 31 March 2017 were used to identify all BTOA injections undertaken in adults in the National Health Service secondary care in England. Patients were followed up longitudinally until death or 31 March 2017. A multivariable regression with a Fine and Gray model adjusting for the competing risk of mortality in addition to age, sex and socioeconomic deprivation was used to identify factors associated with progression to further procedure. Secondary outcomes included serious complications after injection and subsequent surgical site infection.

**Results:**

A total of 19 120 primary injections were performed during the 19-year period in 18 356 patients. Of these 76.5% were female; mean age 62 years (s.d. 10.6); 50.48% underwent further procedure; 22.40% underwent surgery. Median time to further intervention was 412 days (IQR 110–1945). Female sex was associated with increased risk of proceeding to surgery. Serious complication rate following injection was 0.04% (0.01–0.08) within 90 days. Of those proceeding to surgery 0.16% (0.06–0.34) presented with a wound infection within 30 days and 90 days, compared with an overall post-operative wound infection rate of 0.03% (0.02–0.05).

**Conclusions:**

Very low rates of serious complications were identified following BTOA injections performed in secondary care; only one in five patients proceeded to subsequent surgery.

**Clinical trial registration:**

clinicaltrials.gov, https://www.clinicaltrials.gov, NCT03573765

**Rheumatology key messages**

- This large national cohort identified that 50% of cases proceeded to further intervention after intra-articular steroid BTOA injection.
- 22% proceeded to surgery at any time, mostly within a year of injection.
- Very low rates of serious complications of BTOA intra-articular injection in secondary care were found.

## Introduction

Base of thumb osteoarthritis (BTOA) is a common hand condition presenting to primary and secondary care physicians, characterized by pain and reduced function [1–3]. Early treatment options for BTOA include intra-articular steroid injection in addition to splinting and hand therapy [4, 5]. Developing best evidence for hand arthritis is a research priority for patients with hand conditions in the UK [6].

Systematic reviews of available randomized control trials and case series noted that evidence of efficacy of intra-articular steroid injections for BTOA was limited and heterogeneous [7–10]. Smaller single-centre studies have estimated that following BTOA intra-articular steroid injection, only around one-third proceed to surgery [11].

Efficacy aside, research from a recent large US insurance dataset raised concerns that BTOA steroid injections predispose patients to a higher risk of post-operative complications [12]. However, previous studies in other areas of the body have found no evidence to support this finding [13, 14].

Study into the long-term course of treatment and risk of complications within routine clinical care is therefore an important addition to the literature in order to better counsel patients. Observational research offers the opportunity to follow patients for longer after an intervention than clinical trials, and enables rare complications and complications that do not present within a short time frame to be better identified [15].

### Objectives

Our primary aim was to estimate the incidence of further procedures after intra-articular steroid injection for BTOA in adults in the NHS in England. Secondary aims were to identify factors associated with proceeding to further intervention, especially surgery, serious complications and whether having a BTOA injection prior to surgery affected the risk of serious surgical site infection.

## Methods

### Data source

A bespoke pseudonymized extract of individual-level patient data from the NHS Digital Hospital Episode Statistics for Admitted Patient Care (HES APC) dataset was made (1 April 1998 to 31 March 2017). This extract contained all episodes of NHS care associated with BTOA, defined by a validated list of codes [16]. HES APC contains all admissions, including day-case care, for all individuals, and the extract contained all episodes before and after the ‘index’ BTOA episode. The extract contained all episodes of care remunerated by the NHS in England, including independent providers (i.e. private hospitals undertaking procedures on behalf of the NHS on NHS patients). All patient episodes of care within the NHS England system are linked via a patient’s individual NHS number. This enabled linkage of all NHS-funded treatments undertaken and longitudinal follow-up of each patient. The HES APC extract was also linked to the ONS national mortality dataset prior to pseudonymization to identify cause and date of death [17]. The NHS covers the vast majority of health-care provision in England, with only 11% of the population estimated to hold private health-care insurance, and only 13% of all elective surgery being privately funded outside the NHS [18].

### Ethical approval

This study was approved by the University of Oxford Research Services Clinical Trials Research Group (project ID 12787), and the NHS Data Access Advisory Group (DAAG). It was carried out in accordance with the NHS Digital Data Sharing Agreement (DARS-NIC-29827-Q8Z7Q) and registered at clinicaltrials.gov (NCT03573765). Studies using non-identifiable records from Hospital Episode Statistics are exempt from research ethics committee approval. Patients have the right to request that their data is not released by NHS Digital for use by researchers (register a ‘Type 2 opt-out’).

### Population

Patients identified as having a BTOA injection were followed up until death, or censored at the end of the study (31 March 2017) in order to maximize the longitudinal follow-up possible within the dataset. Minimum follow-up was 1 day, to capture all complications including those occurring within the first 24 h post-operatively. Duplicate episodes can occur over the change of financial year, and these were removed.

Exposures and outcomes were defined using previously validated OPCS-4.7 classification for interventions and International Classification of Disease (ICD) version 10 codes for disease (Supplementary Table S1, available at *Rheumatology* online), defined in an initial validation study for identification of all cases of BTOA in secondary care [19–21].

Two further clinical validation studies were undertaken within our institution to look at the patient population defined within the injection cohort, and the validity of identifying surgical subtypes in HES APC. Discussion with clinical coders, NHS Digital and a sample of over 300 patients undergoing injection or surgery within 1 year was undertaken. The injection cohort was confirmed to include patients undergoing injection in theatre, in specialist outpatient injection clinics run by rheumatologists and hand surgeons, and those undergoing injection in the radiology department as an outpatient procedure. The injection validation study showed we were able to identify patients who had undergone a BTOA intra-articular injection with a positive predictive value of 85.8% using our previously validated code list (Supplementary Table S1, available at *Rheumatology* online). In the second clinical validation study, the coding for BTOA surgical subtypes found a positive predictive value of 99% in our Trust within a year’s sample of 104 patients undergoing BTOA surgery, and therefore our code list was considered appropriate.

In order to further characterize the population included, factors associated with the development of BTOA were identified using OPCS and ICD-10 codes (Supplementary Table S2, available at *Rheumatology* online). A past medical history of carpal tunnel syndrome, generalized osteoarthritis, knee osteoarthritis, rheumatoid arthritis, hand or wrist fracture, and oophorectomy was identified if the patient had an episode including the relevant code at any time prior to or within the hospital episode for BTOA injection. To determine socio-economic status, Index of Multiple Deprivation (a Government generated score of relative deprivation based on geographical location within England) was used, and the Charlson Comorbidity Index was used to determine overall combined comorbidity level of each patient at the time of injection or surgery*.* Ethnicity was included as defined by NHS Digital [22–24].

A further procedure undertaken in secondary care was defined as a code for surgery after injection or a second injection in the same hand when calculating incidence rates, survival and regression analysis. In order to identify the ‘worst-case scenario’ of possible patients who may go on to a procedure but have missing laterality codes, a further definition of three or more procedures per person was also included when calculating an estimate of the number of people requiring a further procedure.

To estimate the number of cases proceeding to surgery after injection, surgery was defined as any episode containing the OPCS and ICD codes in Supplementary Table S1, available at *Rheumatology* online, that occurred after injection. Laterality linked injection and surgery was again used in survival analysis and surgical intervention rates.

Serious complications after primary intra-articular injection as identified in hospital admission records were defined as severe infection (septic arthritis, wound infection leading to wound dehiscence or wound debridement) and tendon injury. As these complications were identified from HES APC, the complications required an episode of admission to hospital including as a day-case patient or requiring surgery, occurring in the same hand as the injection within 30 or 90 days of injection (see Supplementary Table S3, available at *Rheumatology* online). Surgical site infection after BTOA surgery for those who had a pre-operative intra-articular steroid injection was defined in the same manner, and compared with the rates seen in all post-operative BTOA surgery within the HES extract. The NHS framework of complications within 30 and 90 days was used to determine the comparative incidence rate [25]. All results with a count of <7 were redacted to reduce the risk of secondary disclosure of data according to the NHS Digital analysis guide [26]. Cox proportional hazard analysis of the factors associated with post-operative complications was planned a priori.

### Statistical methods

We calculated age- and sex-specific incidence rates of surgery using ONS mid-year population estimates [27]. All complications were calculated as a proportion of the sample with 95% confidence intervals (CI). Incomplete records consisted of only 0.74% cases for age, sex, ethnicity and Index of Multiple Deprivation deciles, and were assumed to be missing at random. We therefore did not employ any imputation, but undertook complete case analysis. Laterality code was present in 93.8%; comparison of demographics of those with and without laterality present within their records demonstrated that the patients were comparable (Supplementary Table S4, available at *Rheumatology* online).

Kaplan-Meier analysis was undertaken to identify the trend in time to further intervention or surgery. We identified factors associated with further intervention using a Fine and Gray model to produce both a crude and adjusted sub-hazard ratios (sHR) accounting for the competing risk of mortality [28]. Proportional hazards assumption was tested using Schoenfeld residuals. Age was categorized and the category containing the median age (60–69 years) was used as the baseline category due to the non-linear relationship of age with adverse outcome that did not meet the proportional hazards assumption. Statistical analysis was undertaken using Stata version 15.1. A Poisson distribution was assumed and the delta method was used to calculate confidence intervals for complications.

## Results

### Patient demographics

**Fig. 1:**
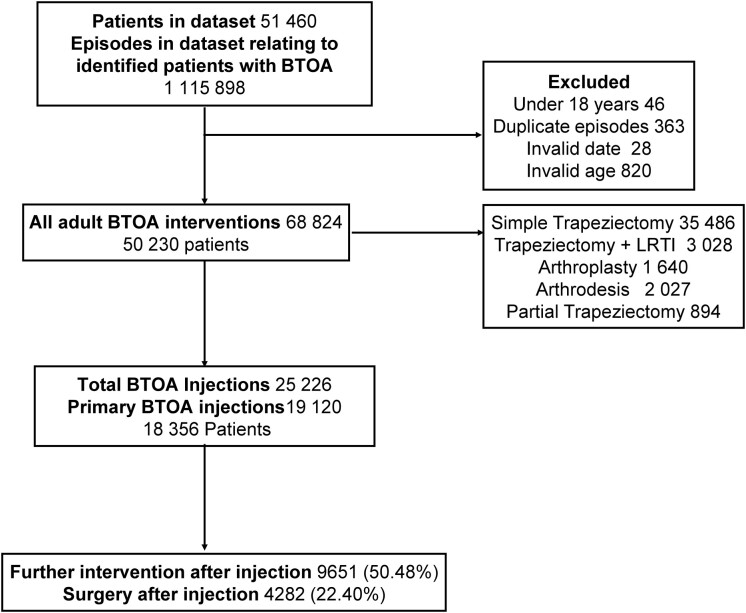
Data processing flow chart

Figure 1 describes the data processing details. In the study period, 19 120 primary BTOA injections (18 356 patients) and 4282 operations were performed on 3863 individuals in English NHS hospitals. Median follow-up time was 5.0 years [interquartile range (IQR) 2.2– 8.8 years]. Of these patients 128 (0.67%) had <30 days of follow-up without an event due to having their primary injection during March 2017; 351 (1.8%) patients had <90 days of follow-up without an event, i.e. due to having primary injection between January and March 2017 (1.40%). A total of 76.5% of patients were female, and the mean age at injection was 62 years (s.d. 10.6). A peak of intervention was observed in women around the peri-menopausal age that was not as prominent in men (Supplementary Fig. S1, available at *Rheumatology* online). In all, 64.7% of patients had a low level of overall comorbidity with a Charlson Comorbidity Index of zero or one. A total of 83% of patients identified themselves as being of a white background, and the socio-demographic distribution of patients undergoing primary BTOA injection was roughly even across the strata. The full demographic profile of patients undergoing primary BTOA injection, further intervention and surgical intervention is shown in Table 1.

**Table 1:** Baseline characteristics of patients undergoing BTOA injection, and those undergoing further intervention post-injection

Age category	All injection patients freq (%)	Any intervention after 1st injection freq (%)	Surgery after 1st injection freq (%)
18–29	49 (0.3)	7 (0.1)	<7^a^
30–39	280 (1.5)	70 (0.7)	23 (0.5)
40–49	1797 (9.4)	804 (8.3)	369 (8.6)
50–59	5975 (31.3)	3051 (31.6)	1443 (33.7)
60–69	6490 (34.0)	3534 (36.6)	1618 (37.8)
70–79	3486 (18.3)	1785 (18.5)	698 (16.3)
>80	1022 (5.4)	398 (4.1)	123 (2.9)
Missing	21 (0.1)	2 (0.02)	<7^a^
Total	19 120	9651	4278
IMD decile			
Least deprived 10%	1651 (8.6)	911 (9.4)	397 (9.3)
Less deprived 10–20%	1805 (9.4)	987 (10.2)	394 (9.2)
Less deprived 20–30%	2119 (11.1)	1033 (10.7)	458 (10.7)
Less deprived 30–40%	2179 (11.4)	1069 (11.1)	468 (10.9)
Less deprived 40–50%	2030 (10.6)	1001 (10.4)	437 (10.2)
More deprived 10–20%	1760 (9.2)	894 (9.3)	394 (9.2)
More deprived 20–30%	1902 (10.0)	918 (9.5)	434 (10.1)
More deprived 30–40%	1844 (9.6)	932 (9.7)	421 (9.9)
More deprived 40–50%	1988 (10.4)	1027 (10.6)	466 (10.9)
Most deprived 10%	1726 (9.0)	836 (8.7)	381 (8.9)
Missing	116 (0.6)	43 (0.5)	28 (0.7)
Total	19 120	9 651	4278
Ethnic group			
Any White background	16 018 (83.8)	8700 (90.2)	3866 (90.4)
Any Asian background	354 (1.85)	140 (1.5)	50 (1.2)
Any Black background	70 (0.4)	22 (0.2)	13 (0.3)
Any mixed background	41 (0.2)	9 (0.1)	<7^a^
Chinese	16 (0.1)	11 (0.1)	<7^a^
Any other ethnic group	96 (0.5)	33 (0.3)	11 (0.3)
Not stated	2233 (11.7)	659 (6.8)	268 (6.3)
Not known	292 (1.0)	77 (0.8)	28 (0.7)
	19 120	9651	4278
Charlson index			
0	7881 (41.2)	4139 (42.9)	1966 (46.0)
1	4486 (23.5)	2474 (25.6)	1078 (25.2)
2	2535 (13.3)	1230 (12.7)	523 (12.2)
3	1538 (8.0)	738 (7.7)	306 (7.2)
4	836 (4.4)	361 (3.7)	128 (3.0)
>=5	1844 (9.6)	709 (7.4)	277 (7.4)
Total	19 120	9651	4278
Comorbidities			
Carpal tunnel syndrome	2123	1107	550
Knee osteoarthritis	2258	1155	515
General osteoarthritis	4102	2092	804
Rheumatoid arthritis	142	30	<7^a^
Wrist fracture	110	36	17
Oophorectomy	661	380	185

a Numbers <7 suppressed in line with NHS Digital disclosure control guidelines – percentages of other groups rounded to prevent secondary disclosure of data [26]. IMD: Index of Multiple Deprivation.

### Trends in further intervention

**Fig. 2:**
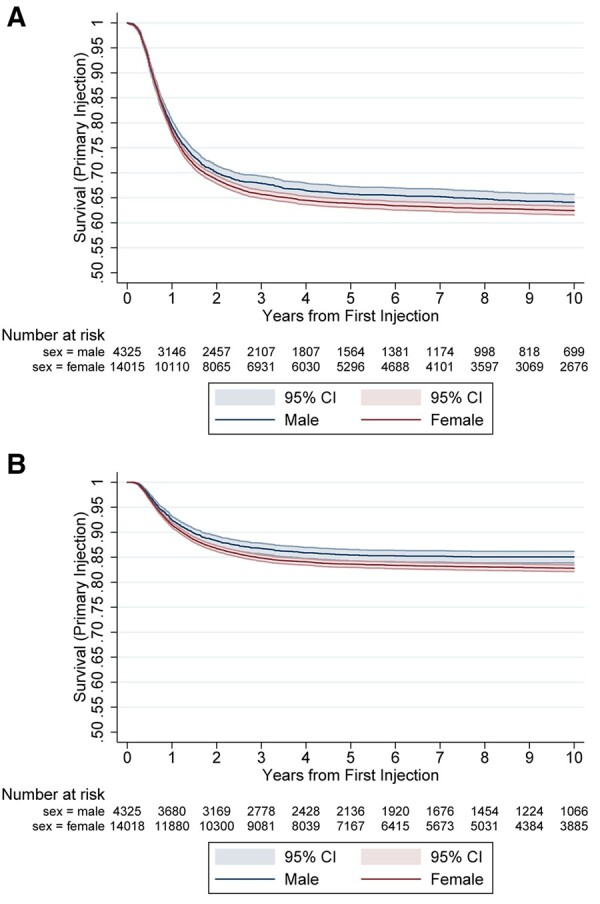
Kaplan-Meier plots divided by sex for risk of (**A**) further procedure and (**B**) surgery after primary BTOA injection

**Fig. 3:**
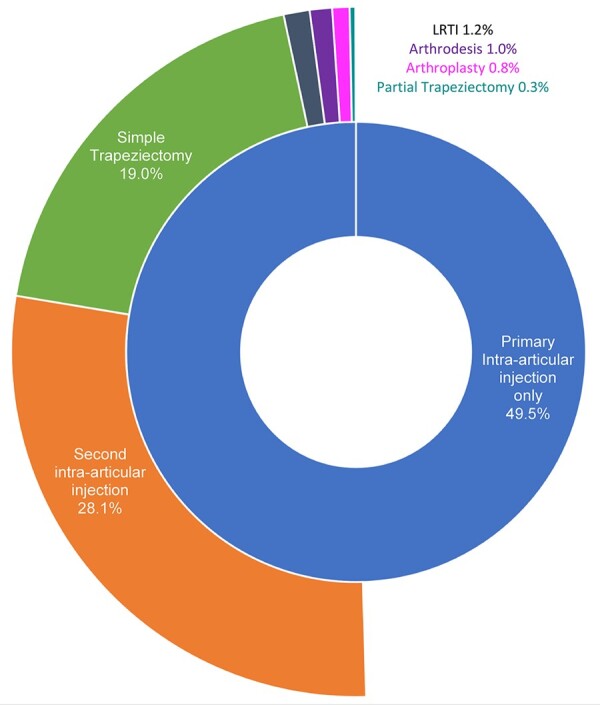
Sunburst representing procedures that occurred following primary BTOA injection

In total, 9651 further interventions were identified after primary BTOA injection in 6461 individuals. The median time to second procedure was 412 days (IQR 110–1945), with an incidence rate of 66.7 per 1000 person-years (95% CI: 65.06, 68.41). A total of 4282 surgeries were undertaken after an injection at any point giving an incidence rate of 22.3 per 1000 person-years (95% CI: 21.51, 23.19). Kaplan-Meier analysis of time to further intervention and surgery is given in Figs. 2a and 2b. The sunburst plot in Fig. 3 illustrates the treatment paths taken in secondary care following primary BTOA injection. The central ring represents all primary intra-articular injections in the cohort, and the outer ring represents the number and type of subsequent interventions undertaken during the follow-up period. The central ring shows that 49.5% of primary injections had no subsequent intervention observed. Of the 50.5% of patients with primary injections who were observed to undergo a further intervention, 28.1% underwent a second intra-articular injection, with simple trapeziectomy being the most common surgical procedure undertaken following BTOA injection.

### Factors associated with further intervention after injection

**Fig. 4:**
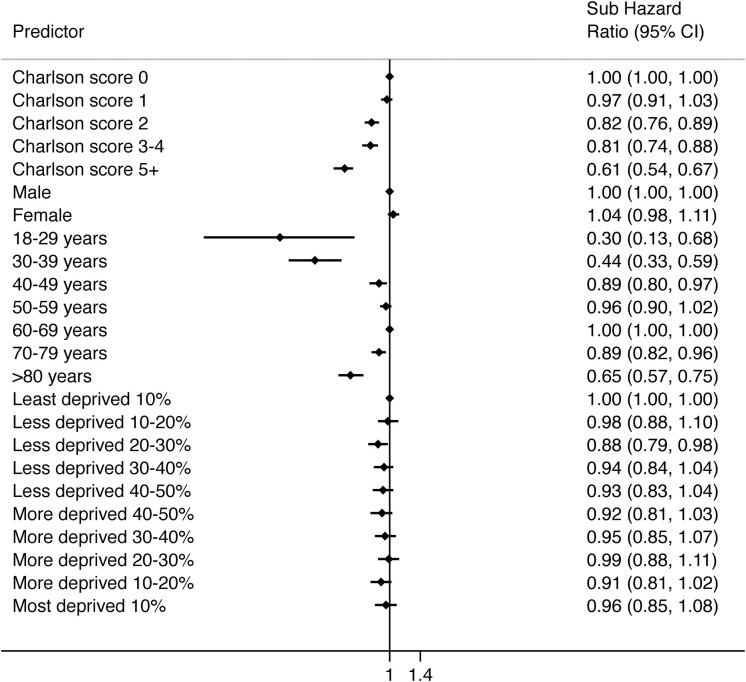
Forest plot of factors associated with proceeding to further intervention following BTOA injection

Crude univariable analysis suggested an association of increased incidence of further intervention with female sex. This was not confirmed in multivariable analysis (Fig. 4) when adjusting for age, comorbidity and socio-economic status. Compared with those in the median age category, patients who were at the extremes of the age range at the time of primary injection had a reduced risk of further intervention that persisted in adjusted analysis [adjusted sHR 0.30 (0.13–0.68) for those age 18–29 years, adjusted sHR 0.44 (0.33–0.59) for those age 30–39 years; Supplementary Table S5, available at *Rheumatology* online]. Increasing levels of comorbidity were associated with reduced incidence of further intervention and there was no association seen between further intervention and socio-economic status.

**Fig. 5:**
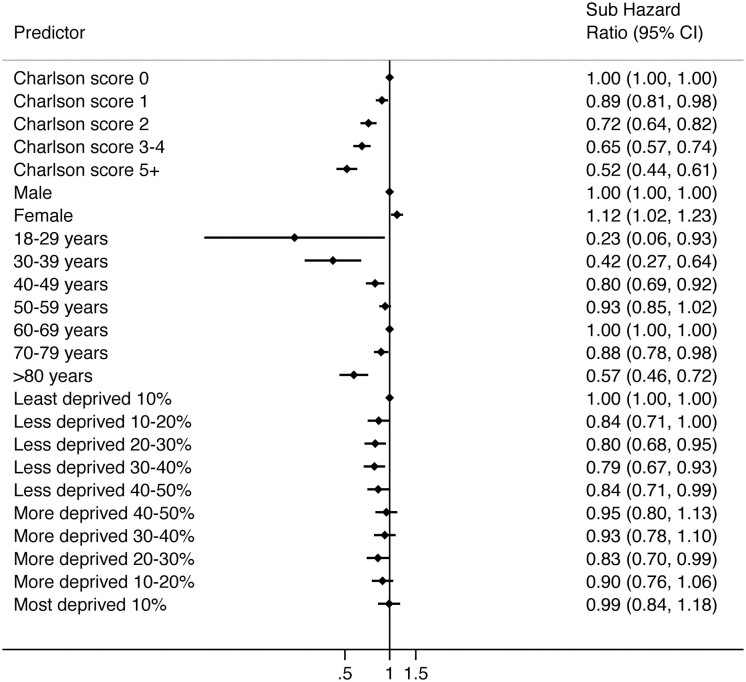
Forest plot of factors associated with proceeding to surgery following BTOA injection

When considering the factors associated with proceeding to surgery after injection, female sex was associated with a 12% increased relative risk within multivariable analysis [adjusted sHR 1.12 (1.02–1.23); Fig. 5; Supplementary Table S6, available at *Rheumatology* online]. As was seen with all further intervention, there was a reduced likelihood of progressing to surgery at the extremes of age, and with increasing comorbidity. No association was found with socio-economic status.

### Complications after injection

There were a very small number of cases identified as complicated by septic arthritis, neurovascular injury, need for wound debridement or tendon repair after primary injection in secondary care. As all absolute numbers were under 7, the true value must be minimized according to NHS Digital analysis rules. This gives a maximum rate of 0.04% (95% CI: 0.01, 0.08) within 30 or 90 days of injection for wound debridement or tendon repair and a rate of 0.04% (95% CI: 0.01, 0.08) for septic arthritis or neurovascular injury at any post-operative time point within this cohort.

### Complications after subsequent surgery

In the 4282 thumbs that underwent intra-articular injection in secondary care prior to undergoing surgery, <7 cases presented with serious surgical site infection within 30 or 90 days. The true value must therefore be minimized, but gives a maximum rate of surgical site infection for those having a pre-operative injection of 0.16% (0.06–0.34). In total, 43 076 BTOA surgeries were identified in the 19-year period, of which only 0.03% presented with serious surgical site infection within 30 or 90 days, respectively.

## Discussion

### Key findings

This large national cohort study observed ∼50% of primary intra-articular BTOA injections in secondary care proceeded to further intervention. The most common further procedure was a repeat injection. One in five patients in the cohort went on to have surgery at a median time of 412 days following injection.

Patients at the extremes of age and with greater levels of comorbidity were observed to be less likely to undergo further injection or progress to surgery. When adjusted for age, social deprivation and comorbidity, female sex was observed to be associated with increased risk of progression to surgery. A very low rate of complications was seen in secondary care following injection, with <4 in 10 000 patients needing hospital treatment for severe infection, neurovascular injury or tendon injury. Although a higher incidence of surgical site infection was seen if patients underwent a pre-operative intra-articular injection at any time prior to surgery, the incidence of serious infection remained below 2 in 1000 for complications within 90 days of surgery.

This study adds to the literature surrounding the incidence of serious complications following intra-articular steroid injections in the hand. Our data are in stark contrast to the rate of post-surgical complications found in US data containing insured and Medicare (state-assisted health care) patients, where 21% of patients sustained any form of complication after BTOA surgery [12]. In their study, undergoing steroid injection prior to surgery increased the odds of a complication by 20%, although no absolute rate of complications was reported for those patients who had pre-operative intra-articular injections. Giladi *et al.* used a wider definition for infection (including any diagnosis of infection or prescription of antibiotics within 6 weeks of surgery), which may explain some of the disparity, since only the most serious complications seen in secondary care are included in our study. A difference in studied populations within a different health-care system may also have an impact.

### Strengths and limitations

Our study contains data from a national source with longitudinal follow-up, enabling patients to be followed within a nationalized health system including if they move to a different health-care provider for subsequent procedures. It includes all patients presenting to a public health-care system that includes patients with a wide age range and range of associated comorbidities and levels of social deprivation. As England in general has a low rate of intervention undertaken in the private sector, this NHS data will capture the majority of health-care activity to produce more generalizable results for the role of interventions for BTOA in a general population. This data identifies trends in patients who may be excluded from clinical trials, and observes patients in health care outside trial centres and health-care settings engaged in research. Results found in our study align with previous work identifying positive responses to intra-articular steroid injection for hand osteoarthritis without serious complications [29, 30].

This study is limited to interactions within secondary care only, where patients have an injection under radiological guidance, in an injection clinic, or in theatre. The patients included are therefore only those undergoing primary injection that is registered within this system. Our validation studies showed that the HES APC dataset includes patients undergoing intra-articular injections under radiological guidance or on a specific injection list, but will not include those being undertaken in traditional secondary care outpatient clinic settings or that had occurred previously in primary care or in interface services. This produces a selection bias in the patients included, but also indicates that patients included here are those who have been referred to secondary care. As the data does not link to primary care, we cannot fully record treatment that has gone before, and this is acknowledged as a limitation.

Similarly, our study only reports complications that are sufficiently severe to present in secondary care, and will not include, for example, minor infections treated with oral antibiotics in primary care. Our data define the risk of serious surgical site infection or significant tendon injury requiring intervention, and can inform the consent process regarding the most serious and most clinically important complications. It must be recognized that as the study is based within secondary care alone, only complications that require an inpatient admission or intervention will therefore be included. Whilst patients could present to any secondary care provider within the NHS in England and this would be detected by linkage through their individual identifier, presentations to primary care are not included. We believe that the low rates identified here are not due to misclassification bias or underreporting, but more that they only include the most serious events. Whilst this study adds to the literature by identifying the rates of the most serious complications within a national cohort, further work is needed to identify other complications that would not require admission or further intervention, for example within primary care observational datasets.

Information regarding a patient’s comorbidities in this study are only collected from HES and therefore may not be as rich as in primary care datasets, but may contain selection bias of the most pertinent comorbidities likely to affect outcome from secondary care intervention. HES APC also does not collect data on the use of orthoses, thus we cannot compare the use of adjuvant splints in this population alongside intra-articular injection, which should be recognized as a limitation. However, because HES APC is an administrative dataset repurposed to enable research, we have undertaken validation studies in order to minimize misclassification of cases. HES APC data has the significant advantage of preventing inclusion bias, as data is collected outside the main research team.

### Future work

Further work is needed to describe the rate of minor infective complications and side effects that would not produce an admitted patient care episode, identifying the rate of complications seen in primary and intermediate care in routinely collected data. Similarly, a large cohort of patients providing additional data complications such as steroid flare and skin depigmentation following injection, and use of orthoses in secondary care would also provide a comprehensive picture of the role of intra-articular injection for BTOA. Replication in other countries would determine whether similar secondary care trends are also seen outside a national health-care system. This study only investigates intra-articular injections overall, and as the NHS only routinely undertakes intra-articular steroid injections, it does not compare with other agents such as hyaluronic acid that are not routinely undertaken in the NHS. A great deal of prior scientific work has focused on comparing the efficacy of the two injections within clinical trials, and future work could compare their efficacy within routine clinical care if both agents are used in one health-care system [31–34]. This study also does not compare between radiologically guided or blind injections, or compare patient-reported outcomes following injection or surgery, and this could be further investigated. Finally, this study found that progression to surgery was more common in women, and further investigation into the reasons for difference in disease progression to surgery between the sexes would enable greater understanding of the factors associated with BTOA disease progression.

## Supplementary Material

keaa925_Supplementary_Data

## Notes

Daniel Prieto-Alhambra and Dominic Furniss co-directed this work

## References

[1] Dahaghin S, Bierma-Zeinstra SM, Ginai AZ et al Prevalence and pattern of radiographic hand osteoarthritis and association with pain and disability (the Rotterdam study). Ann Rheum Dis 2005;64:682–7. 15374852 10.1136/ard.2004.023564 PMC1755481

[2] Moriatis Wolf J, Turkiewicz A, Atroshi I, Englund M. Prevalence of doctor-diagnosed thumb carpometacarpal joint osteoarthritis: an analysis of Swedish health care. Arthritis Care Res (Hoboken) 2014;66:961–5. 24339432 10.1002/acr.22250

[3] Yu D, Peat G, Bedson J, Jordan KP. Annual consultation incidence of osteoarthritis estimated from population-based health care data in England. Rheumatology (Oxford). 2015;54:2051–60. 26163287 10.1093/rheumatology/kev231 PMC4603278

[4] Bahadir C, Onal B, Dayan VY, Gürer N. Comparison of therapeutic effects of sodium hyaluronate and corticosteroid injections on trapeziometacarpal joint osteoarthritis. Clin Rheumatol 2009;28:529–33. 19137353 10.1007/s10067-008-1079-6

[5] Parker S, Riley N, Dean B; Oxford Upper Limb Collaborative. Management of osteoarthritis at the base of the thumb. Bone Joint J 2020;102-B:600–5. 32349588 10.1302/0301-620X.102B5.BJJ-2019-1464.R2

[6] James Lind Alliance. Common Conditions Affecting the Hand and Wrist. 2017. http://www.jla.nihr.ac.uk/priority-setting-partnerships/common-conditons-affecting-the-hand-and-wrist/ (27 April 2020, date last accessed).

[7] Kroon FPB, Carmona L, Schoones JW, Kloppenburg M. Efficacy and safety of non-pharmacological, pharmacological and surgical treatment for hand osteoarthritis: a systematic literature review informing the 2018 update of the EULAR recommendations for the management of hand osteoarthritis. RMD Open 2018;4:e000734. 30402266 10.1136/rmdopen-2018-000734 PMC6203105

[8] Meenagh GK, Patton J, Kynes C, Wright GD. A randomised controlled trial of intra-articular corticosteroid injection of the carpometacarpal joint of the thumb in osteoarthritis. Ann Rheum Dis 2004;63:1260–3. 15361383 10.1136/ard.2003.015438 PMC1754748

[9] Spaans AJ, van Minnen LP, Kon M et al Conservative treatment of thumb base osteoarthritis: a systematic review. J Hand Surg Am 2015;40:16–21. e1–6. 25534834 10.1016/j.jhsa.2014.08.047

[10] Fowler A, Swindells MG, Burke FD. Intra-articular corticosteroid injections to manage trapeziometacarpal osteoarthritis—a systematic review. Hand (N Y). 2015;10:583–92. 26568708 10.1007/s11552-015-9778-3 PMC4641109

[11] Ostergaard PJ, Hall MJ, Dowlatshahi AS, Harper CM, Rozental TD. Thumb carpometacarpal arthritis: prognostic indicators and timing of further intervention following corticosteroid injection. J Hand Surg Am 2020;45:986.e1–986.e9. 32451202 10.1016/j.jhsa.2020.03.025

[12] Giladi AM, Rahgozar P, Zhong L, Chung KC. Corticosteroid or hyaluronic acid injections to the carpometacarpal joint of the thumb joint are associated with early complications after subsequent surgery. J Hand Surg Eur Vol 2018;43:1106–10. 30335596 10.1177/1753193418805391

[13] Pereira LC, Kerr J, Jolles BM. Intra-articular steroid injection for osteoarthritis of the hip prior to total hip arthroplasty: is it safe? A systematic review. Bone Joint J 2016;98-B:1027–35. 27482013 10.1302/0301-620X.98B8.37420

[14] Berthelot JM, Le Goff B, Maugars Y. Side effects of corticosteroid injections: what's new? Joint Bone Spine 2013;80:363–7. 23352513 10.1016/j.jbspin.2012.12.001

[15] Makady A, van Veelen A, Jonsson P et al Using real-world data in health technology assessment (HTA) practice: a comparative study of five HTA agencies. PharmacoEconomics 2018;36:359–68. 29214389 10.1007/s40273-017-0596-z PMC5834594

[16] NHS Digital. Hospital Episode Statistics (HES). 2018. https://digital.nhs.uk/data-and-information/data-tools-and-services/data-services/hospital-episode-statistics (16 September 2020, date last accessed).

[17] NHS Digital. Linked HES-ONS mortality data. 2018. https://digital.nhs.uk/data-and-information/data-tools-and-services/data-services/linked-hes-ons-mortality-data#ons-mortality-data (16 September 2020, date last accessed).

[18] The King’s Fund. The UK private health market. London: King’s Fund, 2014.

[19] Lane JS, Green J, Lam WL, Furniss D, Sudlow C. Can we use routinely collected hospital and GP data for epidemiological study of common hand conditions? A UK Biobank based validation project. Biorxiv 2018; doi: 10.1101/274167.

[20] NHS Digital. National clinical coding standards: OPCS-4 and ICD-10. 2017. https://digital.nhs.uk/services/terminology-and-classifications/clinical-classifications (16 September 2020, date last accessed).

[21] World Health Organization (WHO). International Classification of Diseases, version 10. 2016. https://www.who.int/classifications/icd/icdonlineversions/en/ (16 September 2020, date last accessed).

[22] Quan H, Sundararajan V, Halfon P et al Coding algorithms for defining comorbidities in ICD-9-CM and ICD-10 administrative data. Medical Care 2005;43:1130–9. 16224307 10.1097/01.mlr.0000182534.19832.83

[23] Ministry of Housing, Communities and Local Government. The English indices of deprivation. UK Government, 2018. https://www.gov.uk/government/collections/english-indices-of-deprivation (16 September 2020, date last accessed).

[24] Quan H, Li B, Couris CM et al Updating and validating the Charlson comorbidity index and score for risk adjustment in hospital discharge abstracts using data from 6 countries. Am J Epidemiol 2011;173:676–82. 21330339 10.1093/aje/kwq433

[25] NHS Digital. NHS Outcomes Framework. 2018. https://digital.nhs.uk/data-and-information/publications/ci-hub/nhs-outcomes-framework (16 September 2020, date last accessed).

[26] NHS Digital. Hospital Episode Statistics (HES) Analysis Guide. 2019.

[27] Office for National Statistics (ONS). Estimates of the population for the UK, England and Wales, Scotland and Northern Ireland. 2018. https://www.ons.gov.uk/peoplepopulationandcommunity/populationandmigration/populationestimates/datasets/populationestimatesforukenglandandwalesscotlandandnorthernireland (16 September 2020, date last accessed).

[28] Fine JP, Gray RJ. A proportional hazards model for the subdistribution of a competing risk. J Am Stat Assoc 1999;94:496–509.

[29] Favero M, Ramonda R, Rossato M. Efficacy of intra-articular corticosteroid injection in erosive hand osteoarthritis: infrared thermal imaging. Rheumatology (Oxford). 2017;56:86. 27658905 10.1093/rheumatology/kew333

[30] Favero M, Hoxha A, Frallonardo P et al Efficacy and safety of ultrasound-guided intra-articular glucocorticoid injection in erosive hand osteoarthritis. Pain Med 2020; 10.1093/pm/pnaa261 32914191

[31] Monfort J, Rotés-Sala D, Segalés N et al Comparative efficacy of intra-articular hyaluronic acid and corticoid injections in osteoarthritis of the first carpometacarpal joint: results of a 6-month single-masked randomized study. Joint Bone Spine 2015;82:116–21. 25311256 10.1016/j.jbspin.2014.08.008

[32] Heyworth BE, Lee JH, Kim PD et al Hylan versus corticosteroid versus placebo for treatment of basal joint arthritis: a prospective, randomized, double-blinded clinical trial. J Hand Surg Am 2008;33:40–8. 18261664 10.1016/j.jhsa.2007.10.009

[33] Stahl S, Karsh-Zafrir I, Ratzon N, Rosenberg N. Comparison of intraarticular injection of depot corticosteroid and hyaluronic acid for treatment of degenerative trapeziometacarpal joints. J Clin Rheumatol 2005;11:299–302. 16371798 10.1097/01.rhu.0000191194.39926.c9

[34] Trellu S, Dadoun S, Berenbaum F, Fautrel B, Gossec L. Intra-articular injections in thumb osteoarthritis: a systematic review and meta-analysis of randomized controlled trials. Joint Bone Spine 2015;82:315–9. 25776442 10.1016/j.jbspin.2015.02.002

