# Supplementary material for: Low rate of subsequent surgery and serious complications following intra-articular steroid injection for base of thumb osteoarthritis: national cohort analysis
Source: Rheumatology (Oxford). 2021 Jan 7;60(9):4262–71. doi: 10.1093/rheumatology/keaa925 (PMC8410003; doi:10.1093/rheumatology/keaa925)
Supplement: keaa925_Supplementary_Data [file keaa925_supplementary_data.docx]

**Supplementary Table S1. OPCSv4.7 and ICD-10 codes used to identify intraarticular injection and surgery for base of thumb osteoarthritis**

| **BTOA OPCS codes** | | | **AND** | **BTOA ICD-10 codes** |
| --- | --- | --- | --- | --- |
| W57.2 | alone |  | with | M18.0-18.9 |
| W57.3 | alone |  | or | M15.1-2 |
| W57.8 | **WITH ANY** | Z82.3 |  |  |
| W02.8 |  | Z72.4 |  |  |
| W02.9 |  | Z73.8 |  |  |
| W06.8 |  | Z73.9 |  |  |
| W06.9 |  | Z89.5 |  |  |
| W08.5 |  | Z82.2 |  |  |
| W08.8 |  | Z82.9 |  |  |
| W08.9 |  |  |  |  |
| W13.8 |  |  |  |  |
| W17.8 |  |  |  |  |
| W17.9 |  |  |  |  |
| W28.1 |  |  |  |  |
| W33.8 |  |  |  |  |
| W33.9 |  |  |  |  |
| W43.1 |  |  |  |  |
| W44.1 |  |  |  |  |
| W45.1 |  |  |  |  |
| W45.3 |  |  |  |  |
| W54.1 |  |  |  |  |
| W54.3 |  |  |  |  |
| W56.2 |  |  |  |  |
| W57.2 |  |  |  |  |
| W62.1 |  |  |  |  |
| W62.2 |  |  |  |  |
| W62.8 |  |  |  |  |
| W62.9 |  |  |  |  |
| W63.1 |  |  |  |  |
| W63.2 |  |  |  |  |
| W63.8 |  |  |  |  |
| W63.9 |  |  |  |  |
| W64.1 |  |  |  |  |
| W64.2 |  |  |  |  |
| W64.8 |  |  |  |  |
| W64.9 |  |  |  |  |
| W74.2 |  |  |  |  |
| W74.3 |  |  |  |  |
| W77.5 |  |  |  |  |
| W77.7 |  |  |  |  |
| W77.8 |  |  |  |  |
| W90.3 |  |  |  |  |
| W90.4 |  |  |  |  |
| X38.1 |  |  |  |  |
| X38.2 |  |  |  |  |
| Y65.8 |  |  |  |  |
| Y82.2 |  |  |  |  |

**Supplementary Table S2. OPCS v4.7 classification for interventions, International Classification of Disease (ICD) version 10 codes used to identify covariates for BTOA**

| **Covariates for BTOA** | **ICD-10 or OPCS code** |
| --- | --- |
| Carpal Tunnel Syndrome | G560 (OPCS A651) |
| Knee Osteoarthritis | M17 |
| Generalised Osteoarthrits | M15; M19 |
| Rheumatoid Arthritis | M05 |
| Wrist/Hand fracture | S62 |
| Oophorectomy | (OPCS Q22,Q23, Q43) |

**Supplementary Table S3. OPCSv4.7 and ICD-10 codes used to identify complications following BTOA injection and surgery**

| **Complications** | **code** |
| --- | --- |
| Wound dehiscence | OPCS Z82 + (S60.4 or S42.2 or S42.3 or S42.4) |
| Tendon repair | OPCS Z82 + T67 |
| Wound debridement | OPCS Z82 + (T96.8 or T96.3 or T96.4) |
| Neurovascular injury | ICD code T81.2 |
| Septic Arthritis | ICD M004 M0014 M0024 M0084 M0104 M0114 M0124 M0134 M0144 M0154 M0164 M0184 |

**Supplementary Table S4.** **Comparison of patients with and without laterality code (baseline demographics)**

|  | **With laterality code** | **Without laterality code** |
| --- | --- | --- |
| **Total** | 18013 | 1107 |
| **Sex** |  |  |
| Male | 4241 | 251 |
| Female | 13769 | 855 |
| Missing | 3 | 1 |
|  |  |  |
| **Mean age (SD; years)** | 62.40 (10.57) | 62.67 (10.86) |
| **Charlson index** |  |  |
| 0 | 7459 (41.41) | 422 (38.12) |
| 1 | 4237 (23.52) | 249 (22.49) |
| 2 | 2391 (13.27) | 144 (13.01) |
| 3 | 1439 (7.99) | 99 (8.94) |
| 4 | 776 (4.31) | 60 (5.42) |
| >=5 | 1711 (9.51) | 133 (12.01) |
| missing | 0 | 0 |
|  |  |  |
| **IMD decile** |  |  |
| Least deprived 10% | 1545 | 106 |
| Less deprived 10-20% | 1712 | 93 |
| Less deprived 20-30% | 1998 | 121 |
| Less deprived 30-40% | 2044 | 135 |
| Less deprived 40-50% | 1921 | 109 |
| More deprived 10-20% | 1659 | 101 |
| More deprived 20-30% | 1789 | 113 |
| More deprived 30-40% | 1735 | 109 |
| More deprived 40-50% | 1873 | 115 |
| Most deprived 10% | 1630 | 96 |
| Missing | 18013-17906 | 1107-1098 |
|  |  |  |
| **Ethnic group** |  |  |
| Any white background | 15133 (84.01) | 885 (80) |
| Any Asian background | 317 (1.76) | 20 (1) |
| Any Black background | 62 (0.35) | <7* |
| Any mixed background | 39 (0.22) | <7* |
| Chinese | 0 | <7* |
| Any other ethnic group | 95 (0.53) | <7* |
| Not stated | 2056 (11.42) | 177 (16) |
| Not known | 93 (0.52) | 16 (1) |
| missing | 0 | 0 |
|  |  |  |
| **Co variates** |  |  |
| Carpal Tunnel Syndrome | 2011 | 112 |
| Knee Osteoarthritis | 2138 | 120 |
| General Osteoarthritis | 3864 | 238 |
| Rheumatoid Arthritis | 137 | 5 |
| Wrist Fracture | 106 | 4 |
| Oophorectomy | 631 | 30 |
| *Numbers less than 7 suppressed in line with NHS Digital disclosure control guidelines- percentages of other groups rounded to prevent secondary disclosure of data (29) | | |

**Supplementary Table S5. Risk of any intervention after BTOA injection using subhazard ratio (sHR) accounting for the competing risk of mortality**

|  | **Crude sHR** | **95% CI** | **Adjusted sHR** | **95% CI** |
| --- | --- | --- | --- | --- |
| **Sex** |  |  |  |  |
| Female | 1.07 | 1.01 to 1.14 | 1.04 | 0.98 to 1.11 |
| **Age category** |  |  |  |  |
| 18-29 years | 0.32 | 0.14 to 0.72 | 0.30 | 0.13 to 0.68 |
| 30-39 years | 0.47 | 0.35 to 0.63 | 0.44 | 0.33 to 0.59 |
| 40-49 years | 0.95 | 0.86 to 1.04 | 0.89 | 0.80 to 0.97 |
| 50-59 years | 1.00 | 0.94 to 1.07 | 0.96 | 0.90 to 1.02 |
| 60-69 years | 1 (reference) | 1 (reference) | 1 (reference) | 1 (reference) |
| 70-79 years | 0.85 | 0.79 to 0.92 | 0.89 | 0.82 to 0.96 |
| Over 80 years | 0.60 | 0.53 to 0.69 | 0.65 | 0.57 to 0.75 |
|  |  |  |  |  |
| **Charlson Comorbidity Index** |  |  |  |  |
| 0 | 1 (reference) | 1 (reference) | 1 (reference) | 1 (reference) |
| 1 | 0.97 | 0.91 to 1.03 | 0.97 | 0.91 to 1.03 |
| 2 | 0.81 | 0.75 to 0.88 | 0.82 | 0.76 to 0.89 |
| 3-4 | 0.80 | 0.73 to 0.87 | 0.81 | 0.74 to 0.88 |
| 5+ | 0.59 | 0.54 to 0.66 | 0.61 | 0.54 to 0.67 |
| **Index Multiple Deprivation** |  |  |  |  |
| Least deprived 10% | 1 (reference) | 1 (reference) | 1 (reference) | 1 (reference) |
| Less deprived 10-20 | 0.98 | 0.88 to 1.10 | 0.98 | 0.88 to 1.10 |
| Less deprived 20-30% | 0.88 | 0.79 to 0.98 | 0.88 | 0.79 to 0.98 |
| Less deprived 30-40% | 0.94 | 0.84 to 1.05 | 0.94 | 0.84 to 1.04 |
| Less deprived 40-50% | 0.92 | 0.82 to 1.03 | 0.93 | 0.83 to 1.04 |
| More deprived 10-20% | 0.90 | 0.80 to 1.01 | 0.92 | 0.81 to 1.03 |
| More deprived 20-30% | 0.94 | 0.84 to 1.05 | 0.95 | 0.85 to 1.07 |
| More deprived 30-40% | 0.98 | 0.87 to 1.10 | 0.99 | 0.88 to 1.11 |
| More deprived 40-50% | 0.89 | 0.80 to 1.00 | 0.91 | 0.81 to 1.02 |
| Most deprived 10% | 0.92 | 0.82 to 1.04 | 0.96 | 0.85 to 1.08 |
| Adjusted subhazard ratio represents results of multivariable regression analysis adjusted for all other factors included (i.e. sex, age, charlson comorbidity index and index of multiple deprivation) | | | | |

|  | **Crude sHR** | **95% CI** | **Adjusted sHR** | **95% CI** |
| --- | --- | --- | --- | --- |
| **Sex** |  |  |  |  |
| Female | 1.15 | 1.05 to 1.27 | 1.12 | 1.02 to 1.23 |
| **Age category** |  |  |  |  |
| 18-29 years | 0.25 | 0.06 to 1.00 | 0.23 | 0.06 to 0.93 |
| 30-39 years | 0.47 | 0.30 to 0.73 | 0.42 | 0.27 to 0.64 |
| 40-49 years | 0.88 | 0.76 to 1.01 | 0.80 | 0.69 to 0.92 |
| 50-59 years | 1.00 | 0.91 to 1.10 | 0.93 | 0.85 to 1.02 |
| 60-69 years | 1 (reference) | 1 (reference) | 1 (reference) | 1 (reference) |
| 70-79 years | 0.82 | 0.73 to 0.91 | 0.88 | 0.78 to 0.98 |
| >80 years | 0.51 | 0.41 to 0.63 | 0.57 | 0.46 to 0.72 |
|  |  |  |  |  |
| **Charlson Comorbidity Index** |  |  |  |  |
| 0 | 1 (reference) | 1 (reference) | 1 (reference) | 1 (reference) |
| 1 | 0.90 | 0.82 to 0.98 | 0.89 | 0.81 to 0.98 |
| 2 | 0.72 | 0.64 to 0.82 | 0.72 | 0.63 to 0.82 |
| 3-4 | 0.65 | 0.57 to 0.74 | 0.65 | 0.57 to 0.74 |
| 5+ | 0.51 | 0.44 to 0.60 | 0.52 | 0.84 to 1.18 |
| **Index Multiple Deprivation** |  |  |  |  |
| Least deprived 10% | 1 (reference) | 1 (reference) | 1 (reference) | 1 (reference) |
| Less deprived 10-20 | 0.85 | 0.72 to 1.01 | 0.84 | 0.71 to 1.00 |
| Less deprived 20-30% | 0.80 | 0.68 to 0.94 | 0.80 | 0.68 to 0.95 |
| Less deprived 30-40% | 0.80 | 0.67 to 0.93 | 0.79 | 0.67 to 0.93 |
| Less deprived 40-50% | 0.83 | 0.70 to 0.98 | 0.84 | 0.71 to 0.99 |
| More deprived 10-20% | 0.92 | 0.77 to 1.09 | 0.95 | 0.80 to 1.13 |
| More deprived 20-30% | 0.90 | 0.76 to 1.06 | 0.93 | 0.78 to 1.10 |
| More deprived 30-40% | 0.81 | 0.68 to 0.97 | 0.83 | 0.70 to 0.99 |
| More deprived 40-50% | 0.87 | 0.74 to 1.03 | 0.90 | 0.76 to 1.06 |
| Most deprived 10% | 0.94 | 0.79 to 1.11 | 1.00 | 0.84 to 1.18 |
| Adjusted subhazard ratio represents results of multivariable regression analysis adjusted for all other factors included (i.e. sex, age, charlson comorbidity index and index of multiple deprivation) | | | | |

**Supplementary Table S6. Risk of surgery after BTOA injection using sub-hazard ratio (sHR) accounting for the competing risk of mortality**

**Supplementary Figure S1. Age and sex specific incidence of primary BTOA injections, England, 1998-2017.**
